# Prevalence of multiple sclerosis in Liguria region, Italy: an estimate using the capture–recapture method

**DOI:** 10.1007/s10072-021-05718-w

**Published:** 2021-11-24

**Authors:** M. Ponzio, A. Tacchino, D. Amicizia, M. F. Piazza, C. Paganino, C. Trucchi, M. Astengo, S. Simonetti, D. Gallo, A. Sansone, G. Brichetto, M. A. Battaglia, F. Ansaldi

**Affiliations:** 1 Scientific Research Area, Italian Multiple Sclerosis Foundation, Genoa, Italy; 2 A.Li.Sa, Liguria Health Authority, Genoa, Italy; 3 Department of Health Sciences, University of Genoa, Genoa, Italy; 4 Liguria Digitale S.P.A, Genoa, Italy; 5 AISM Rehabilitation Centre Liguria, Italian Multiple Sclerosis Society, Genoa, Italy; 6 Department of Life Sciences, University of Siena, Siena, Italy

**Keywords:** Multiple sclerosis, Prevalence, Capture–recapture method, Italy

## Introduction

Multiple sclerosis (MS) is the most common nontraumatic disease of the central nervous system [1, 2] that causes permanent disability in young adults. During its clinical course, various neurodegenerative and autoimmune processes are known to be taking place; however, its etiology is unknown. Females are more commonly affected than males and the sex ratio has increased over the past decades [3].

MS affects 2.5 million people worldwide [4]. There are significant geographical differences with the highest prevalence occurring in Northern Europe and North America (over 100/100,000) and lower prevalence rates observed in Asia and Africa (< 5/100,000) [5, 6]. Prevalence and incidence estimates for MS are often based on clinical records provided by MS centers. This approach could produce a risk of underestimating the proportion of patients who do not attend clinical centers with a consequent underestimation of the true prevalence. This leads to biased estimates even after data aggregation of larger geographical areas (e.g., regional, national) [6, 7].

In recent years, there has been a growing interest in developing methods based on health administrative databases to estimate the prevalence of chronic diseases, in order to lead to more precise and reliable incidence and prevalence estimates [8]. Different algorithms have been developed in various clinical contexts, according to literature and available data sources [9–12]. Unfortunately, even data based on administrative sources might lead to the underestimated prevalence and incidence rates; thus, whenever possible, the extent of undercount should be at least assessed, and possibly corrected.

In Italy, a country considered a high-risk area for MS, several epidemiological studies have been regionally and locally conducted with different methodologies, resulting in a wide range of prevalence estimations ranging from 122 to 232 cases per 100,000. A recent study, based on these reference data, shows that Italy is a high prevalence country for MS with about 109,000 people affected and an estimated prevalence of approximately 179 per 100,000 persons [7]. However, despite the high prevalence, precise estimates of epidemiology are missing for some Italian regions, such as the Liguria region. Population-based administrative (health claims) datasets offer a potentially efficient, cost-effective, and generalizable approach to such studies. In fact, recently increasing interest in the possibility to use the administrative databases to obtain more precise and reliable estimates of the prevalence of many diseases including MS [13]. As a note in several areas of epidemiology, the data based on different sources (as administrative claims) might lead to underestimated prevalence rates, therefore the extent of undercount should be at least assessed, and possibly corrected. Establishing prevalence in specific regions can contribute to the understanding of etiologic factors and inform decisions related to resource allocation and access to MS care and supports [14–16].

The objective of this study was the assessment of MS prevalence in the Liguria region using routinely collected healthcare data. In particular, we validated the algorithm used to identify MS cases by administrative data sources, we evaluated the dependence and potential heterogeneity of the sources and we estimated the MS prevalence on December 31, 2017, in the Liguria region including the proportion of undetected cases.

## Materials and methods

### Area under investigation

Liguria is a small north-western Italian region, covering an area of 5,416 km^2^ and bordering France to the West, Piedmont to the North, Emilia-Romagna, and Tuscany to the East and the Mediterranean Sea to the South. Prevalently mountainous (65.1%), it is administratively divided into four provinces, Genoa, Imperia, La Spezia, and Savona. The reference population was 1,551,541 (746,461 males and 805,080 females; density: 286.5/km^2^; data taken at December 31, 2017), and it is one of the oldest Italian populations worldwide; the mean age is 48.9 versus 45.0 years and 28.5% of the population is over 65 [17].

### Data Sources

The Liguria Health Authority (Azienda Ligure Sanitaria, A.Li.Sa) provided databases of administrative healthcare data (AHD) related to the Liguria region. Specifically, AHD databases, or data warehouses, are a regional service that collects hospital discharge records (HDRs), the flow of outpatient visits, and pharmaceuticals. The HDRs include patient demographic data, admission and discharge dates, discharge status, main and secondary discharge diagnoses, and diagnostic/therapeutic procedures [18]. Data about the chronic diseases were achieved through the Liguria Chronic Condition Data Warehouse platform [19]

Data sources were: disease-specific payment exemptions from a copayment to health care (a-EXE), drug prescription records (b-DPR), and hospital discharge records (c-HDR). We made a record linkage using the tax code as a unique personal identification code, previously anonymized.

From the clinical database of the AISM Rehabilitation Service of Liguria (AISM-Rehab) only composed of records of people with MS (PwMS), we extracted a random sample of 500 records of PwMS specifically resident in Liguria. The sample was used as the gold standard (subjects with a definitive diagnosis) to test the accuracy of the case-finding algorithm. The AISM-Rehab was a good benchmark because it follows a significant proportion of MS patients living in the Liguria region in all stages of the disease. Anonymization was performed by using the same algorithm as for routinely collected healthcare data to allow data linkage.

### Ascertainment

The cases were extracted from AHD databases of the Liguria using a case-finding algorithm already previously used [9, 13, 20]. From the databases, individuals with a diagnosis of MS not registered in the Regional Health Service (RHS) were filtered out. Subjects who met at least one of the selection criteria were considered cases, i.e., individuals with at least one active payment exemption for MS at December 31, 2017, or at least one drug prescription for at least one drug specific for MS, or one hospital discharge with MS diagnosis.

### Statistical analysis

Demographic characteristics are reported using descriptive statistics. The two age groups used were those with age < 50 and those with age ≥ 50, these age groups approximately referred to the mean sample aged.

The accuracy of the case-finding algorithm was assessed, using The AISM-Rehab data as the gold standard, by sensitivity, specificity, positive and negative predictive values. The corresponding 95% confidence intervals (CIs) were calculated based on the exact binomial distribution [21].

The capture–recapture method [22, 23] was used to estimate the number of PwMS in Liguria. In epidemiology, the capture–recapture approach attempts to estimate or adjust for the extent of incomplete ascertainment using information from overlapping lists of cases from different sources. This method provides an estimation of the affected population and is particularly useful when the investigator has clearly incomplete data from two or more sources [24, 25]. In this case, we used the 3-source capture–recapture approach, including the 3 incomplete data sources of MS patients. Dependence between sources was assessed by calculating the odds ratio (95% CI) between each pair of sources, as proposed by Wittes [26]. Source dependence was modeled by adding the corresponding interaction term to the model. The significance of the interaction was assessed using likelihood ratio statistics.

Seven hierarchical log-linear models were fit to the data: 1 model assuming independence among the three data sources, 3 models of one pairwise interaction, and 3 models of two pairwise interactions. The absence of any third-order interaction is the basic assumption of the capture–recapture model [23]. Thus, the log-linear model can estimate the number of MS patients not identified by any of the three sources (hidden subjects) and consequently the total population of MS patients. Log-linear models were fit separately for the overall prevalence of MS cases and by age and sex.

The best-fitting model was determined by goodness-of-fit statistics and the parsimony principle. We applied different information criteria for model selection, including the deviance (*G*^2^), Akaike information criterion (AIC), and the Bayesian information criterion (BIC) [27]. *G*^2^ is a distribution chi-square statistic that measures the level of adjustment of observed data with the proposed model. The smaller the value of *G*^2^, the better the adjustment. The AIC and BIC were calculated as follows: AIC = *G*^2^ − [2 × (*df*)] (1) and BIC = *G*^2^ − [ln(Nobs/2*π*)] × (*df*) (2). In Eq. (1) and (2), *G*^2^ is the likelihood ratio statistic associated with the fit of any model to the data and df denotes the degrees of freedom of the model. In Eq. (2), ln(Nobs) is the natural logarithm of the number of parameters in the model. The model with the smallest AIC and BIC value was selected.

Ninety-five percent goodness-of-fit CIs were calculated based on the likelihood ratio to allow asymmetric intervals and avoid underestimation of the upper and lower limits [28].

The crude prevalence rate was based on the number of subjects with MS in the Liguria region on 31 December 2017, using the 2017 resident population of Liguria [29] (i.e., 1,551,541 inhabitants) as the denominator. The crude rates were normalized to the 2017 Italian population using the direct method [29]. For ascertainment-corrected prevalence rate, the numerator was the number of cases estimated by the chosen capture–recapture model, and the denominator was again the Liguria’s population in 2017. A Poisson distribution was used to calculate 95% confidence intervals (95% CIs) for prevalence estimates. All prevalence rates were produced also stratified by sex and age class.

## Results

Between January 1, 2012, and December 31, 2017, a total of 1,551,541 subjects registered in RHS and alive on December 31, 2017, in Liguria and in the A.Li.Sa database constitutes the study cohort.

### Data matching

Totally 3140 subjects with MS were identified, aggregating the three sources and eliminating any duplicate case that could have occurred. Among them, 65.1% were females (sex ratio 1.86). The mean age was 51.8 years (± 14.1 years). No age differences were present between genders (*p* = 0.084). By stratifying the patients in two age classes (younger < 50 and older ≥ 50 years), we observed 46.6% younger (< 50 years) and 53.4% older (≥ 50 years). Relevant differences among sources in the proportion of captured females were not observed (65.5%, 67.1%, and 64.4% of females listed by a-EXE, b-DPR, and c-HDR, respectively). Conversely, the c-HDR claim tended to capture older subjects (55.0% PwMS with age ≥ 50 years vs. 44.2% for b-DPR and 42.3% for a-EXE, respectively).

**Fig. 1:**
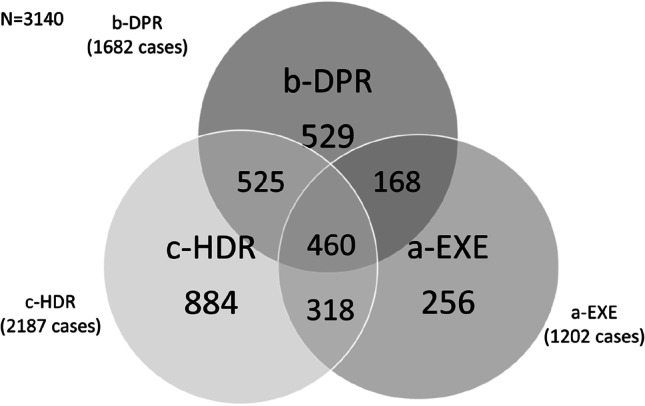
Venn diagram illustrates the distribution of subjects identified by administrative claims: payment exemption (a-EXE), prescription of specific disease drug (b-DPR), and hospital discharge record (c-HDR)

The distribution of subjects identified by administrative claims was reported in the Venn diagram (Fig. 1). In particular, 69.7% (2187 cases) had at least one MS-related hospitalization, 1682 cases (53.6%) had at least 1 prescription for an MS-specific and 38.3% (1202 cases) had an MS-specific payment exemption. Overall, the matching indicates that interactions occurred among sources. Among 3140 MS cases, 1664 (53.0%) are flagged from one source, 1011 (32.2%) from two sources and 460 (14.6%) from three sources. The Wittes odds ration highlighted the negative dependence between b-DPR and c-HDR (OR = 0.30; 95% CI: 0.25–0.39, *p* < 0.001) and c-HDR and a-EXE (OR = 0.69; 95% CI: 0.59–0.80, *p* < 0.001), no dependence was observed between b-DPR and a-EXE (OR = 0.92; 95% CI: 0.79–1.06, *p* = 0.243).

### The accuracy of the case-finding algorithm

From the random sample of 500 PwMS who attended AISM-Rehab, 29 subjects were excused because the tax code was uncorrected, consequently, 471 were used for the analysis. After linking the administrative cohort (subjects identified by case-finding algorithm) and the true-positive reference cohort (AISM-Rehab), we observed that 382 individuals were present in both cohorts, and 80 individuals with a defined MS diagnosis were not included in the administrative cohort. Validation confirmed a MS event in 382 participants, resulting in sensitivity of 82.7% (95% CI: 78.9–86.0%), specificity of 100%, PPV 100%, and NPV 99.9%. Eighty PwMS not identified by algorithm (false-negative) were more frequently females than those identified (80.0% vs. 63.4%, *p* = 0.004), with higher mean age (60.5 (± 16.9) years vs. 53.3 (± 12.2) years, *p* < 0.0001), more frequently secondary progressive form (61.5% vs. 31.3%, *p* < 0.001), higher disease duration (21.0 (± 10.6) years vs. 13.0 (± 9.4) years, *p* < 0.0001) and with higher disability level (EDSS score: 4.9 (± 2.1) vs. 4.2 (± 2.1), *p* = 0.0031).

### C–R estimation

The preliminary results of the capture–recapture method for all samples are shown in Table 1. Capture–recapture utilized the overlap between the lists (Fig. 1) to determine the degree of under ascertainment for the raw count of 3140 and thus provided an estimate of the total MS population. The log-linear model that best fit the data included dependence among the sources. The *p*-values indicate that there were significant differences between the saturated model (the eighth model, abc ab bc ac) and all other reduced models. The fifth model (abc ab bc) was the best-fitting model, with the smallest *G*^2^, AIC, and BIC values. According to these results, it has been estimated that about 890 PwMS were not identified by any of the data sources, and consequently the actual estimated number of PwMS is 4030 (95% CI, 3903 to 4176). To verify potential heterogeneity of the sources, we ran new models including, in addition to dependence among sources, also age classes as covariate (as reported before, c-HDR claim tended to capture older subjects). When considering also the dependency with the variable of catchability (i.e., age class), the model with the lowest G^2^ (177.8), AIC (167.8), and BIC (167.4) provided an estimate of 3992 pwMS. This model included two interactions between sources (abc ab bc) and interactions between sources and the variable of catchability (a*age class, b*age class, and c*age class). Finally, we carry out an analysis stratified by gender and age. For sex, the most parsimonious and best-fitting models were the models of 2 pairwise interactions. In particular, 1068 females (best fit model for female *G*^2^ = 44.0, AIC = 42.0, BIC = 42.0) and 403 males (best fit model for male G^2^ = 13.6, AIC = 11.6, BIC = 11.7) were not identified by our three sources. Also for age, the most parsimonious and best-fitting models were the models of 2 pairwise interactions. A total of 247 younger PwMS (best fit model for younger *G*^2^ = 5.4, AIC = 3.4, BIC = 3.4) and 736 older PwMS (best fit model for older G^2^ = 37.1, AIC = 35.1, BIC = 35.1) were not identified by our three sources.

**Table 1:** Preliminary log-linear models fitted to the MS patients registered in the 3 data sources and the estimated number of hidden MS subjects

Model	*K*	df	G^2^	AIC	BIC	*P*-value	Estimated no. of hidden	Estimated total	95% CI
abc	3	3	130.7	124.7	124.9	< 0.001	553	3693	3626–3766
abc ab	4	2	93.4	89.4	89.4	< 0.001	646	3786	3705–3876
abc ac	4	2	101.2	97.2	97.2	< 0.001	668	3808	3721–3906
abc bc	4	2	129.8	125.8	125.8	< 0.001	524	3664	3581–3758
abc ab ac	5	1	42.0	40.0	40.0	< 0.001	890	4030	3903–4176
abc ab bc	5	1	91.6	89.6	89.6	< 0.001	711	3851	3725–4000
abc ac bc	5	1	97.0	95.0	95.0	< 0.001	806	3946	3783–4147
abc ab bc ac	6	0	0	0	0	1	1963	5103	4621–5742

a, disease-specific payment exemptions from copayment to health care (EXE); b, drug prescription records (DPR); c, hospital discharge records (HDR); *K*, number of parameters; *df*, degrees of freedom; *G*^*2*^, deviance; *AIC*, Akaike Information Criterion; *BIC*, Bayesian information criterion; *CI*, confidence interval

### Prevalence of MS

The overall crude MS prevalence rate was 202.4 cases per 100,000 (95% CI 195.3–209.5); 146.8/100,000 in men (95% CI 138.1–155.5), 253.9/100,000 in women (95% CI 242.9–264.9); 194.1/100,000 in people < 50 years old (95% CI 184.2–204.1) and 212.8/100,000 in ones ≥ 50 years old (95% CI 202.7–223.0).

Capture–recapture analyses estimated an additional 852 cases of MS, indicating that 21.3% of cases may have been missed. With the capture–recapture adjustment, the overall MS prevalence increased to 259.0 per 100,000 person-years (95% CI 250.9–267.0). Table 2 reports the MS prevalence rates crude, standardized for the Italian population, and adjusted for capture–recapture method and stratified by sex and age.

**Table 2:** Prevalence rates per 100,000 of MS people among Liguria region residents, 2017, overall and by sex and age

	Crude rate (95% CI)	*Standardized rate (95% CI)	Capture–recapture (C–R)	
			No. of hidden cases	C–R rate (95% CI)
Overall	202.4 (195.3–209.5)	200.9 (193.9–208.0)	852	259.0 (250.9–267.0)
Female	253.9 (242.9–264.9)	258.6 (247.3–269.9)	480	313.5 (301.3–325.7)
Male	146.8 (138.1–155.5)	143.4 (134.8–151.9)	403	200.8 (190.7–211.0)
< 50 years	194.1 (184.2–204.1)	192.3 (182.4–202.1)	288	226.9 (216.2–237.7)
≥ 50 years	212.8 (202.7–223.0)	211.0 (200.9–221.1)	689	306.2 (294.0–318–4)

^*^Directly adjusted to the Italian population (2017)

## Discussion

The case-finding algorithm to capture people with MS from routinely collected healthcare data used in our study found an observed crude prevalence of MS in Liguria on December 31, 2017, of 202.4 cases per 100,000 inhabitants (95% CI: 195.3–209.5). After linkage to clinical data, the algorithm showed a sensitivity of 82.7%, with 21.3% of MS cases undetected on capture–recapture models. Previous studies, using similar algorithms, presented with the same or higher sensitivity in MS (85.0–99.0%) [13]. However, the capture–recapture method prevalence estimate of 259.0 per 100,000 inhabitants (95% CI: 250.9–267.0) suggests that this region constitutes a high-risk area for MS [30]. The capture–recapture method with log-linear modeling indicated the existence of source dependence in the lists. It is likely that some negative dependence exists among the three sources used for this study as has been suspected prior to analysis. In other words, appearing on one claim may decrease a PwMS’s chance of being on the other claim. For example, we can assume that the subjects treated with disease-modified drugs (b-DPR claim) tend to have fewer relapses and consequently to have fewer hospital admissions (c-HDR claim).

The administrative case-finding algorithm identified approximately 79% of prevalent MS cases. The accuracy analysis showed that the algorithm does not capture 80 MS cases. The reason for this lack of capture could be explained by the features of the 80 not captured subjects (more secondary progressive form, higher disease duration, and disability level). These subjects could not be in disease-modified treatment or have no hospital discharge record (for no relapses) or have another type of exemption (i.e., low household income exemption) due to an overlap between payment exemptions as possible in Italian National Health Service. This observation is in line with the characteristics of our standard reference source (AISM Rehabilitation Service of Liguria) that follows a significant proportion of patients in a more advanced stage of the disease that consequently often no longer attend the clinical center. The percentage of the cases captured by the RC method seems to cover precisely the share of the hidden cases that escape the sensitivity of the algorithm.

The prevalence rate reported by this study is higher than the unique available data (Italian MS Barometro of 2017; 2850 MS cases corresponding to a prevalence rate of 184 cases per 100,000 inhabitants) [31] in the same geographic area, but estimated by extrapolating prevalence information adjusted by the last published incidence and mortality indexes [7].

We think that our prevalence rate is more corrected because the extent of under-count was assessed. As Hook and Regal reported, a major limitation of the capture–recapture methods, when applied in the epidemiological contest, regarding the impossibility of formally establishing whether any estimate is unbiased [32]. For this reason, it is fundamental to take precautions against the risk of obtaining biased estimates, verifying that the underlying assumptions of the methods are at least plausible in any contest. The first assumption is that the target population is closed. We can say that the study period and the geographic area were the same for all of the sources, therefore this assumption can only be satisfied to a reasonable degree. The second assumption is that false-positive subjects should not be present on any list [33]. In our study, it is assumed that all the listed individuals have MS. In agreement with this assumption, the accuracy analysis reported no false positives after the link between the case-finding algorithm and random sample of individuals with a diagnosis of MS extracted from clinical records of AISM-Rehab. The third assumption is that the capture sources are independent. This represents the most important limitation in capture–recapture methodology as it produces a downward bias in any maximum likelihood estimate [26, 32]. However, we sought to determine and resolve these problems by using log-linear regression to model interactions between samples. Finally, the fourth assumption is that for any single source each case in the population has the same probability of ascertainment, although any two sources may differ in this probability [32]. In contrast, we can reasonably hypothesize that each list tends to preferentially cover different subsets of the population. The age class was identified as a variable of heterogeneous catchability. We observed that considering age class as a heterogeneity source in the log-linear model, improvements of the goodness of fit of the model were obtained. So, the selected model included the variable of heterogeneous catchability and gave an estimate of 3992 cases, which was slightly lower than the model including dependencies between sources only.

## Conclusion

Our study provided, for the first time, an estimated prevalence rate of PwMS in the Liguria region taken to account the extent of undercount. The results showed a high prevalence of PwMS in this region. The capture–recapture method can only give a range of the true number in the population, but the derived estimates are useful for health planning purposes, as they attempt to correct for undercount inherent in administrative data collections.

The use of capture–recapture methods with administrative datasets provides a readily available and relatively cheap method for estimating the true dimension of disease in a population.

We think that the capture–recapture methodology should be used as a cost-effective tool for epidemiological research because it yields a more realistic picture of the impact of the disease and represents a good alternative to the community-based study design for estimating the prevalence of many diseases.
